# Using deep learning to classify pediatric posttraumatic stress disorder at the individual level

**DOI:** 10.1186/s12888-021-03503-9

**Published:** 2021-10-28

**Authors:** Jing Yang, Du Lei, Kun Qin, Walter H. L. Pinaya, Xueling Suo, Wenbin Li, Lingjiang Li, Graham J. Kemp, Qiyong Gong

**Affiliations:** 1 Huaxi MR Research Center (HMRRC), Department of Radiology, Functional and Molecular Imaging Key Laboratory of Sichuan Province, West China Hospital of Sichuan University, Chengdu, 610041 China; 2 Department of Radiology, Chongqing University Cancer Hospital, School of Medicine, Chongqing University, Chongqing, China; 3 Department of Psychiatry and Behavioral Neuroscience, University of Cincinnati, Cincinnati, OH 45219 USA; 4 Department of Biomedical Engineering, School of Biomedical Engineering & Imaging Sciences, King’s College London, London, SE5 8AF UK; 5 Mental Health Institute, the Second Xiangya Hospital of Central South University, Changsha, 410008 Hunan China; 6 Liverpool Magnetic Resonance Imaging Centre (LiMRIC) and Institute of Life Course and Medical Sciences, University of Liverpool, Liverpool, L9 7AL UK; 7 Research Unit of Psychoradiology, Chinese Academy of Medical Sciences, Chengdu, Sichuan China

**Keywords:** Deep learning, Posttraumatic stress disorder, Graph measure, Topological properties, Classification Psychoradiology, Psychoradiology

## Abstract

**Background:**

Children exposed to natural disasters are vulnerable to developing posttraumatic stress disorder (PTSD). Previous studies using resting-state functional neuroimaging have revealed alterations in graph-based brain topological network metrics in pediatric PTSD patients relative to healthy controls (HC). Here we aimed to apply deep learning (DL) models to neuroimaging markers of classification which may be of assistance in diagnosis of pediatric PTSD.

**Methods:**

We studied 33 pediatric PTSD and 53 matched HC. Functional connectivity between 90 brain regions from the automated anatomical labeling atlas was established using partial correlation coefficients, and the whole-brain functional connectome was constructed by applying a threshold to the resultant 90 * 90 partial correlation matrix. Graph theory analysis was used to examine the topological properties of the functional connectome. A DL algorithm then used this measure to classify pediatric PTSD vs HC.

**Results:**

Graphic topological measures using DL provide a potentially clinically useful classifier for differentiating pediatric PTSD and HC (overall accuracy 71.2%). Frontoparietal areas (central executive network), cingulate cortex, and amygdala contributed the most to the DL model’s performance.

**Conclusions:**

Graphic topological measures based on fMRI data could contribute to imaging models of clinical utility in distinguishing pediatric PTSD from HC. DL model may be a useful tool in the identification of brain mechanisms PTSD participants.

**Supplementary Information:**

The online version contains supplementary material available at 10.1186/s12888-021-03503-9.

## Background

Posttraumatic stress disorder (PTSD) is a delayed and lasting dysfunctional response to psychological stress. Patients usually have a long illness with recurring symptoms, often complicated by comorbidities such as substance abuse, depression, anxiety disorder, aggressive behavior, self-injury and suicide, as well as medical complications such as chronic pain and infection, cardiovascular disease and increased risk of dementia [1, 2]. The overall burden of disability and premature death caused by PTSD is therefore high [3]. Children are more vulnerable to PTSD than adults, being 12–25% more likely to develop depression, suicidal behavior and cognitive impairment [4]. The etiology and neuropathology of this complex disease are still not clear [5], and accurate prognosis suffers from the lack of reliable biomarkers.

Two decades ago it was hoped that neuroimaging-based biomarkers would prove diagnostically and prognostically effective in a number of neuropsychiatric diseases. This hope has not yet been realized, as research has revealed an increasingly complex picture of subtle, distributed brain changes varying with individual clinical characteristics. Neuroimaging biomarkers capable of distinguishing PTSD from non-PTSD subjects have received attention [6–9], and two recent studies obtained good results using resting-state fMRI [7, 9]. The brain is a highly interconnected network, and the development of psychiatric illness appears increasingly linked to dysfunctional integration of networks between the cortex and subcortical regions. Many studies have therefore taken whole-brain network metrics and used them as input to a single subject classification [10–12]. Recent advances in psychoradiology, allow the direct noninvasive characterization of brain network topology in neuropsychiatric patients [13–15] and advances in graph-based theoretical analysis have enabled quantification of the whole brain’s topological properties [16, 17], revealing a ‘small-world’ organization (characterized by both high local specialization and high global integration between brain regions) [18, 19], whose networks are anatomically and functionally disrupted in psychiatric disease [20]. We used graph-based analysis to investigate the disrupted topology of the functional brain connectome in PTSD, which throws some light on the pathogenesis of pediatric PTSD as well as yielding potential biomarkers of the disease [21].

‘Deep learning’ (DL) describes a group of representation-learning methods that can automatically identify the optimal representation from the raw data with high order complexity and abstraction [22]. A number of studies have applied DL techniques to the classification of psychiatric disorders based on anatomical brain images obtained by MRI [23] or functional MRI images [24] or by combining structural and functional neuroimaging data [25, 26]. Also, DL has yielded promising results in medical image analysis [27, 28], and may yield higher classification accuracy than alternative methods such as support vector machines (SVM) [29, 30]. It can use the trained network to calculate the low-dimensional code of individual brain features quickly and effectively, so as to deduce the deep structure and further characterise the complex nonlinear relationships [31, 32]. There is precedent in PTSD for nonlinear responses to e.g. therapy [33], and it seems reasonable to expect that the complexity of functional and structural patterns in the pathophysiology might be beyond what traditional linear methods (e.g., PCA, sparse learning) can explain [34].

To date, most studies of neuroimaging-based predictors of classification have examined adult patients or mixed pediatric and adult samples. However, biomarkers may be differentially expressed in pediatric and adult patients [35], and studying patients early in their illness course with limited psychotropic exposure can minimize the confounding effects of illness course and medication. Here we confined ourselves to pediatric patients and age-matched controls.

Recent studies of other neuropsychiatric diseases have used novel graph-based analytic methods as input for single-subject classification [36, 37]. To extend these, we combined them with DL, by following a DL-based dimension reduction phase by an SVM classification phase [34]. We hypothesized (i) that the use of DL would enhance the effectiveness of graph-based metrics derived from resting-state fMRI data. Given previous reports that the central executive network (CEN), amygdala, and parietal gyrus are important siters of dysregulation areas in PTSD [21, 38], we also hypothesized (ii) that these regions would make the greatest contributions to classification performance.

## Methods

### Participants

The participants are survivors of a massive earthquake in the Sichuan Province of western China in 2008. From a total of 4200 earthquake survivors screened between January and August 2009, we selected participants who (i) physically experienced the earthquake, (ii) personally witnessed death, serious injury, or the collapse of buildings, (iii) were younger than 18 years of age, (iv) had an intelligence quotient > 80 and (v) had no diagnosis of PTSD prior to the earthquake. Each participant was interviewed and screened using the PTSD checklist (PCL) [39], and the Clinician-Administered PTSD Scale (CAPS) was completed when PCL scores were ≥ 35 [40]. The subjects were considered eligible for inclusion in the PTSD group with a CAPS score of ≥50. Those with PCL scores < 30 was considered eligible as non-PTSD controls [41]. This yielded a total of 161 potential PTSD patients and 99 non-PTSD. In these subjects, the presence/absence of PTSD and psychiatric comorbidities were confirmed by the Structured Clinical Interview for DSM-IV (SCID; Diagnostic and Statistical Manual of Mental Disorders, Fourth Edition [42]). Both the children and their parents were interviewed, and the information from parents was combined with the psychiatrist to support the diagnosis.

Exclusion criteria were: (i) history of depression, bipolar or psychotic disorder, or neurologic disorder (*n* = 42), (ii) contraindication to MR imaging (*n* = 30), (iii) treatment with psychiatric medications within 2 months before recruitment for MRI scanning (*n* = 24), (iv) unavailability of key data (*n* = 12); (v) left handedness (*n* = 10); (vi) CAPS score > 35 but < 50 (*n* = 8) [41], and (vii) history of brain injury (*n* = 7). With these exclusions to obtain a relatively homogeneous sample, we recruited for the present study 33 drug-free first-episode pediatric PTSD and a demographically matched group of 53 healthy control (HC) subjects who did not develop PTSD.

This study was approved by the Research Ethics Committee of the West China Hospital of Sichuan University. Each child’s guardian provided written, informed consent, and children provided assent prior to participation.

### Data acquisition

A resting-state fMRI dataset was acquired using a 3 T magnetic resonance system (GE EXCITE, Milwaukee, WI) with an eight-channel phased array head coil. The participants were instructed to keep their eyes closed and to think of nothing in particular during the acquisition. The sequence parameters were repetition time/echo time (TR/TE) 2000/30 ms; flip angle 90°; 30 axial slices per volume; 5 mm slice thickness (no slice gap); matrix 64 × 64; field of view (FOV) 240 × 240 mm^2^; voxel size 3.75 × 3.75 × 5 mm^3^. A total of 200 volumes were collected for each subject.

### Data preprocessing

The image data was preprocessed by SPM12 (http://www.fil.ion.ucl.ac.uk/spm). The first 10 time points were discarded to avoid instability of the initial MRI signal. After correction for intravolume acquisition time delay and head motion, the images were spatially normalized to a 3 × 3 × 3 mm^3^ Montreal Neurological Institute 152 template and then linearly detrended and temporally bandpass filtered (0.01–0.08 Hz) to remove low-frequency drift and high-frequency physiological noise. Finally, the global signal, the white matter signal, the cerebrospinal fluid signal, and the motion parameters (three translational and three rotational parameters) were all regressed out [43]. According to the record of head motions within each fMRI run, all participants whose head motion exceeded 1.0 mm of translation or 1.0° of rotation in any direction were excluded. We also calculated the mean frame-wise displacement (FD) for two groups, and there was no difference in the mean FD between these two groups [44].

### Network construction and topological properties

The network was constructed using GRETNA (http://www.nitrc.org/projects/gretna/) [45, 46]. We applied a wide range of sparsity (S) thresholds to all correlation matrices. The value of S was chosen to ensure that thresholded networks were estimable for the small-worldness scalar and the small-world index (σ) was > 1.0. The range of our S thresholds was set to 0.05 < S < 0.40 with an interval of 0.01 [45, 47]. For each network metric, the area under the curve (AUC) was calculated, which provides a summarized scalar for the topological characterization of brain networks independent of a single threshold selection. The AUC metric has been proven to be sensitive in the detection of topological alterations of brain networks.

First, the automated anatomical labeling (AAL) atlas [48] was used to divide the whole brain into 90 cortical and subcortical regions of interest, and each was considered a network node. Next, the mean time series was acquired for each region. The partial correlations of the mean time series between all pairs of nodes (representing their conditional dependencies by excluding the effects of the other 88 regions) were considered the edges of the network [45, 49]. This process resulted in a 90 × 90 partial correlation matrix for each subject, which was converted into a binary matrix (i.e., adjacency matrix) according to a predefined threshold (see below for the threshold selection), where the entry a_ij_ = 1 if the absolute partial correlation between regions i and j exceeds the threshold and a_ij_ = 0 otherwise [45].

For the brain networks at each sparsity level, we calculated both global and node network metrics. The global metrics examined included small-world parameters (for definitions see [47]) including the clustering coefficient C_p_, characteristic path length L_p_, normalized clustering coefficient γ, normalized characteristic path length λ, and small-worldness σ, as well as network efficiency parameters (for details see [50]), including the local efficiency E_loc_ and global efficiency E_glob_. We calculated L_p_ as the harmonic mean distance between all possible pairs of regions to address the disconnected graphs dilemma [51]. The node metrics examined included the node degree, efficiency, and betweenness centrality [52]. Finally, global and nodal network topological properties were included to establish a 277-dimensional graphic feature vector, where features 1–7 were global properties (C_p_, L_p_, γ, λ, E_loc_, E_glob_) and features 8–277 were three nodal properties (degree, betweenness, efficiency) of 90 AAL regions.

### Machine learning model

A two-stage classification pipeline was implemented to differentiate PTSD from HC, in which a feedforward multi-layer neural network was adopted as the initial stage for dimensionality reduction [31]. A strength of this approach is that the neural network can thereby obtain a higher-order (non-linear) representation of the features. A deep neural network facilitates the extraction of optimal low-dimensional representations without requiring expert feature engineering knowledge. The hierarchical network architecture enables dimensionality reduction level by level, and the training process updates network parameters iteratively to automatically optimize low-dimensional representations in the output layer [22]. The two-stage pipeline has been reported to outperform traditional machine learning and feature engineering methods in an application to predicting autism development in at-risk infants [34]. Accordingly, we used a two-stage pipeline with some novel graphic features.

SVM was performed as the second stage to individually discriminate PTSD from HC [53]. This has been widely applied in neuroimaging machine learning studies, and performs well [54]. By finding the hyperplane maximizing the margin between binary classes in the feature space, SVM can learn the classification strategy from a training set and use it to predict individual validation sample. Here we fed the resulting low-dimensional features into a binary linear SVM classifier. During the training process at the SVM stage, 5-fold nested cross-validation was performed to find the optimal hyperparameter *C* from *C* = {10^−3^, 10^−2^, 10^−1^, 1, 10^1^, 10^2^, 10^3^, 10^4^} via grid search. Once the optimal hyperparameter for each fold was determined, SVM was trained again with the whole training set and evaluated on the validation set.

The whole two-stage classification pipeline was trained and evaluated with 10-fold stratified cross-validation. In this scheme the participants were divided into 10 non-overlapping partitions, each with the same proportion of patients and HC. In each one of the 10 iterations of the cross-validation, 9 partitions were used as the training set to train the SVM model, and then the trained model was used to obtain predictions in the remaining partition. These predictions were used to calculate the performance metrics (balanced accuracy, specificity, and sensitivity), and since the test set was not part of the training process, the resulting values were unbiased. The reported performance in each case is the mean value across the cross-validation iterations. All these machine learning analyses were programmed using Python language (Version: 3.8, https://www.activestate.com/products/python/), where the neural network was implemented in the Pytorch library [55], and the SVM was implemented based on LIBSVM [56] in the Scikit-Learn library [57]. The first step of DL model was described in detail in Additional file 1. To estimate the significance of the machine learning model, we performed a nonparametric permutation test to calculate the *p* value for the balanced accuracy [58]. This involved repeating the classification procedure 1000 times with different random permutations of the group labels. We then counted the number of times the balanced accuracy was higher for the permuted labels than the real labels, and divided this number by 1000 to calculate the *p* value.

### Feature contribution to the classification performance

Identifying the features with the highest discriminative power in classifying performance can yield clinical/pathophysiological implications beyond mathematical model performance as assessed in conventional neuroimaging machine learning studies. For the novel two-stage pipeline in DL, we tried to find such discriminative patterns from the dimensionality reduction stage. We extracted all the weight matrices *W* = {*W*_1_, *W*_2_. *W*_3_, *W*_4_} connecting consecutive layers *L* = {*l*_1_, *l*_2_, *l*_3_, *l*_4_, *l*_5_} from the fine-tuned network, where *l*_*i*_ denotes the *i-*th layer and *W*_*i*_ denotes the weight matrix connecting *l*_*i*_ and *l*_*i* + 1_. As described elsewhere [34], each node’s contribution in a given layer *i* was estimated from the weight matrix *W*_*i*_. We started from *l*_4_ and worked backward, keeping nodes with the greatest contributions whose summed contributions represent more than 50% of the weight contribution in *l*_4_. Next, node contributions in *l*_3_ were estimated using a partition of weight matrix *W*_3_ restricted to those surviving nodes in *l*_4_. This calculation was propagated backward until we reached the *l*_1_ where contributions of raw features are available. The resulting top 10 features with the greatest contributions were reported.

**Fig. 1:**
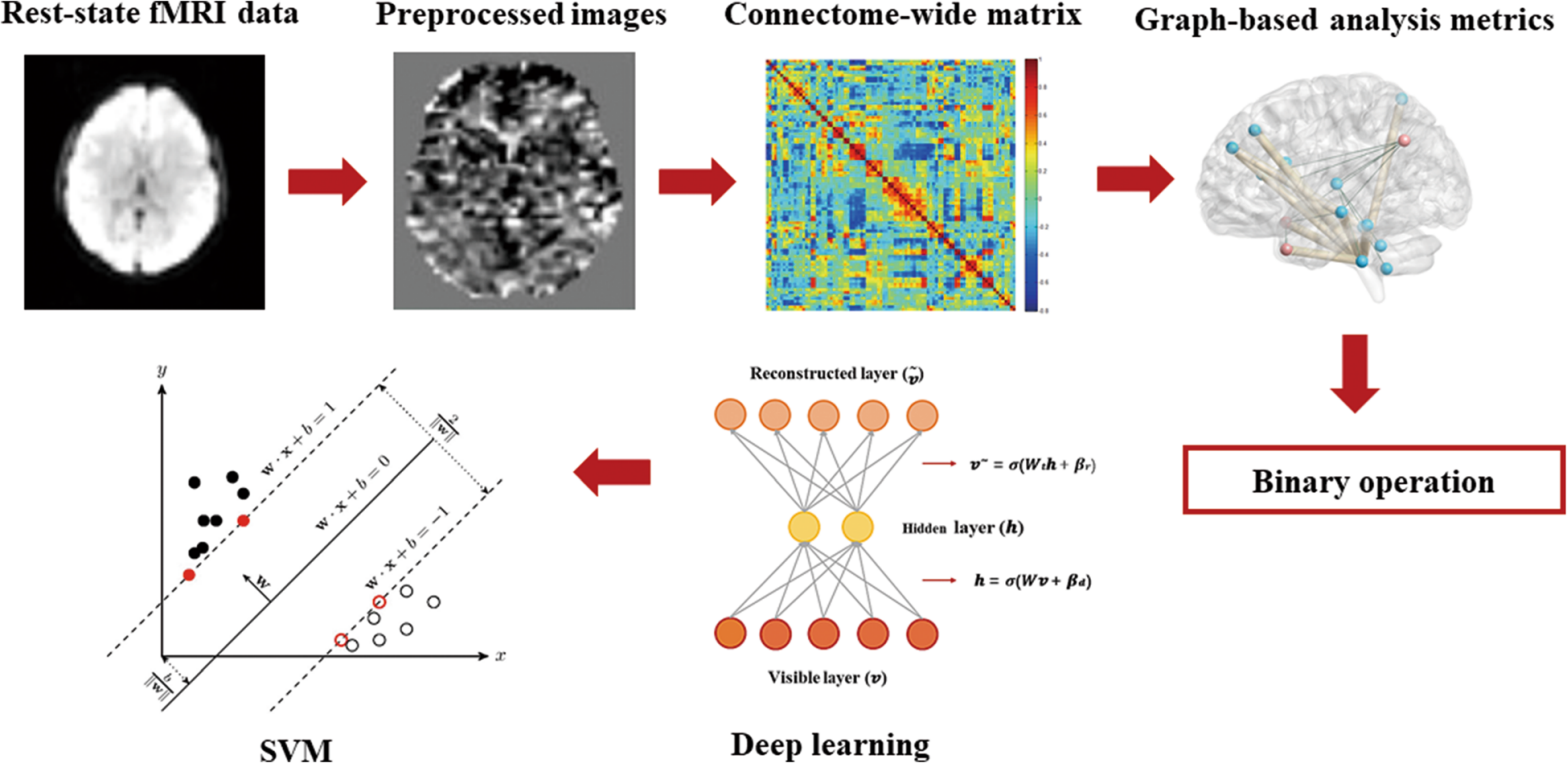
Overview of the employed classification approach showing the main steps of the pipeline. The raw images were preprocessed, and then the whole brain functional connection matrix was calculated to obtain the graphic topological attributes. Finally, the deep learning model was used to classify the groups

**Fig. 2:**
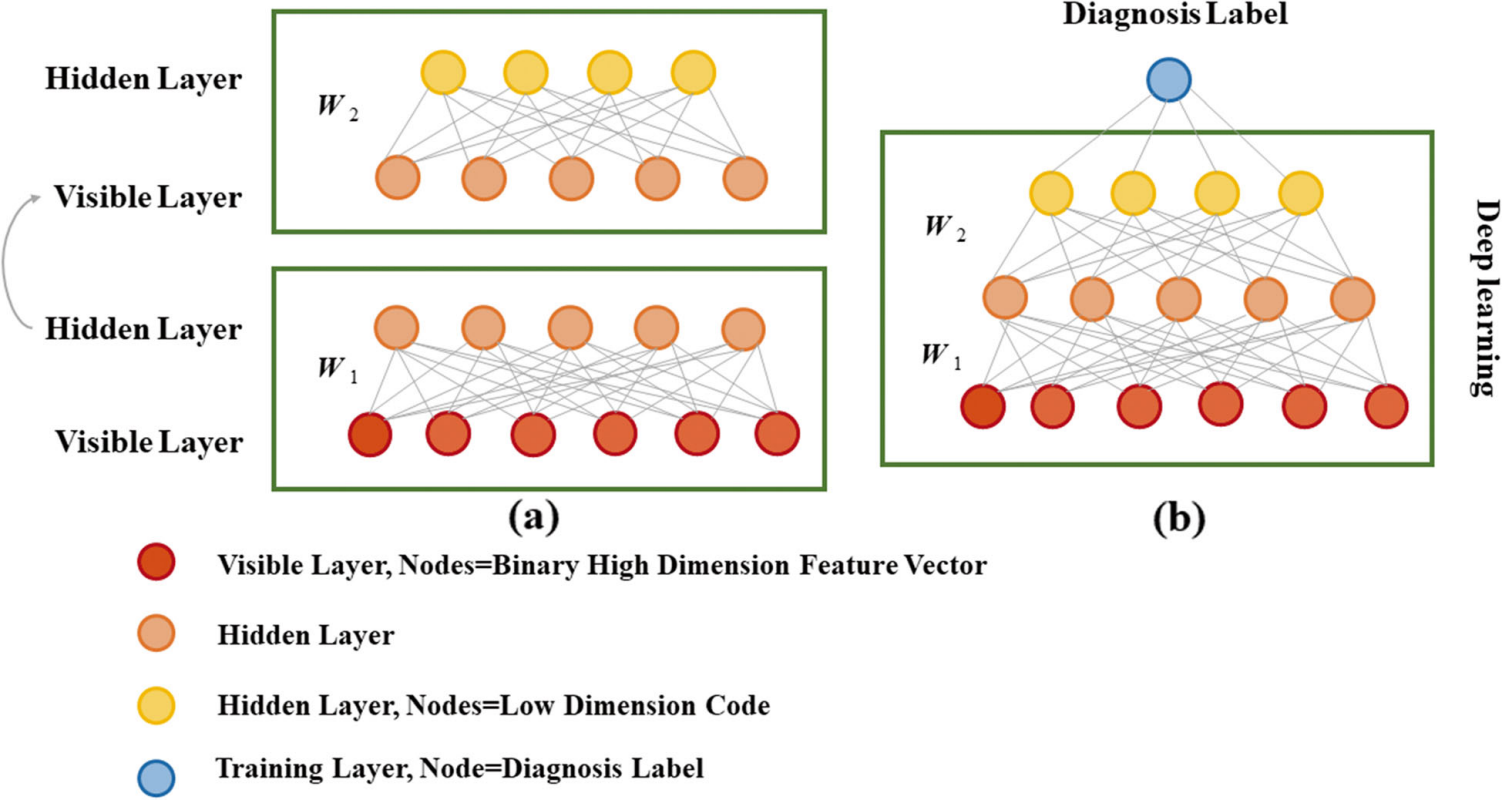
Deep network training. (**a**) An unsupervised step is first performed that sequentially trains individual autoencoders (AE). (**b**) The supervised step stacks the initialized AEs (thus creating the deep network) and then adds one additional layer for the supervised training only (the training label layer) which contains the binary diagnosis label for each binary high-dimension feature vector in the training population [38]

Figure 1 shows an overview of the classification approach showing the main steps in the pipeline. Figure 2 shows the deep network training model. The Supplementary Figure illustrates this process, using a simple example of a 3-layer network; see [34] for details.

### Other statistical methods

The statistical significance of between-group differences in demographic and clinical characteristics was tested by the two-tailed two-sample t test (continuous variables) or the two-tailed Pearson Chi-square test (categorical variables).

## Results

### Demographic and clinical characteristics

There were no significant differences in age, gender, education between pediatric PTSD and HC (*p* > 0.05; Table 1).

**Table 1:** Demographic and clinical characteristics of participants ^a^

Variables	PTSD	HC	***p*** value
Sample size	33	53	–
Age (years) ^b^	14.3 ± 3.3	15.0 ± 2.3	*p* = 0.235 ^c^
Age at trauma (years) ^b^	12.3 ± 1.8	13.9 ± 2.3	*p* = 0.554 ^c^
Gender (male/female)	13/20	26/27	*p* = 0.381 ^d^
Handedness (right/left)	33/0	53/0	–
Education (years)	8.8 ± 2.9	9.5 ± 2.4	*p* = 0.138 ^c^
Time since trauma (months) ^b^	10.5 ± 1.5	13.3 ± 1.4	*p < 0.001* ^c^
PCL	55.7 ± 5.8	23.8 ± 2.9	*p* < 0.001 ^c^
CAPS	65.5 ± 6.6	–	–

^a^Data are presented as mean ± standard deviation

^b^Age defined at the time of MRI scanning

^c^*p* by two-tailed two-sample t test

^d^*p* by two-tailed Pearson Chi-square test

*Abbreviations*: *PTSD* posttraumatic stress disorder, *HC* healthy control, *PCL* PTSD checklist, *CAPS* clinician-administered PTSD scale

### Classification performance

The single-subject classification of pediatric PTSD and HC using graph-based topological metrics was assessed for accuracy, sensitivity and specificity at 10-fold cross-validation. The average accuracy of classification was 71.2 ± 12.9%, the average sensitivity was 59.7 ± 21.9% and the average specificity was 82.7 ± 13.9% in the DL model (*p* < 0.001).

### Regions with greatest contribution to single subject classification

**Fig. 3:**
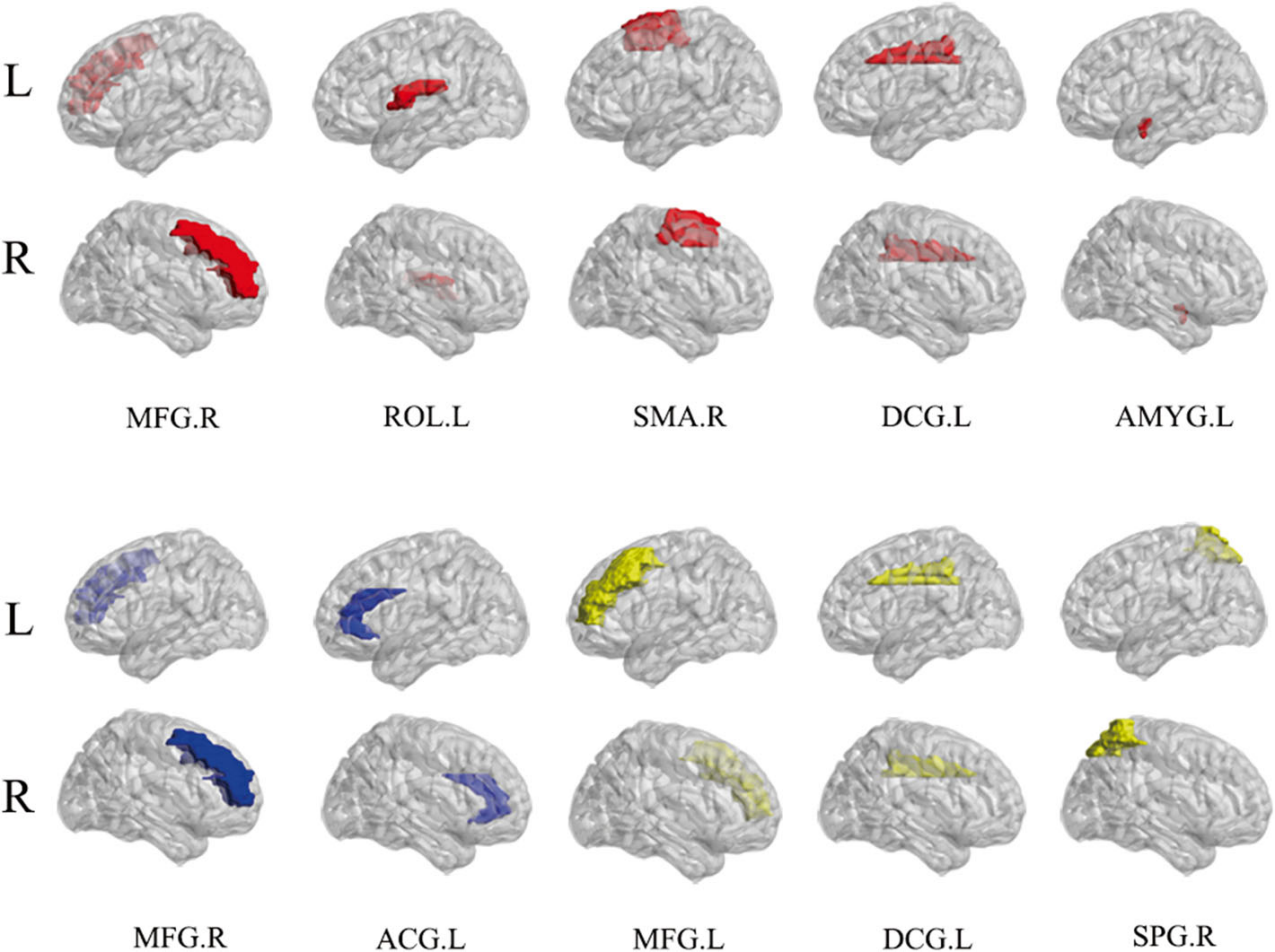
Regions providing the greatest contribution to single subject classification of patients and controls. The nodes (brain regions) were mapped onto the cortical surfaces using the BrainNet Viewer package (http://www.nitrc.org/projects/bnv). For brain regions, red represents the nodal betweenness, blue represents the nodal efficiency, and yellow represents the nodal degree. Abbreviation: DCG, median cingulate and paracingulate gyri; ROL, Rolandic operculum; AMYG, amygdala; ACG, anterior cingulate and paracingulate gyri; MFG, middle frontal gyrus; SMA, Supplementary motor area; SPG, superior parietal gyrus; R, right hemisphere; L, left hemisphere

To identify the classification pattern in patient and HC group, we investigated feature contributions to the non-linear dimensionality reduction in patient group in the DL model. The 10 features with the highest contribution values across the DL models are reported in Table 2 and represented graphically in Fig. 3. These regions were mainly located in frontoparietal areas, with some spread to subcortical regions such as the anterior cingulate cortex, median cingulate cortex, and amygdala.

**Table 2:** Top 10 most relevant topological properties of brain regions for Deep Learning classification analysis ^a^

Topological property	Brain regions	Contributions
Nodal betweenness	Middle frontal gyrus R	0.0079
Nodal betweenness	Amygdala L	0.0078
Nodal betweenness	Supplementary motor area R	0.0077
Nodal betweenness	Rolandic operculum L	0.0076
Nodal degree	Middle frontal gyrus R	0.0075
Nodal degree	Superior parietal gyrus L	0.0074
Nodal efficiency	Anterior cingulate and paracingulate L	0.0072
Nodal degree	Median cingulate and paracingulate L	0.0072
Nodal betweenness	Median cingulate and paracingulate L	0.0071
Nodal efficiency	Middle frontal gyrus R	0.0071

^a^All brain regions are from AAL (automated anatomical labelling)

*Abbreviation*: *R, L* right, left hemisphere

## Discussion

We set out to classify between pediatric PTSD and HC using the DL model applied to graphic topological measures, and then explored the regions making the greatest contribution to classification performance. Consistent with our first hypothesis, we found that using topological properties in DL we could distinguish PTSD from HC at the individual level with significant accuracy. This supports the emerging notion that graphic topological properties based on resting-state functional neuroimaging data can be a powerful tool for characterizing brain disorders at the level of the individual [21]. Such methods have achieved 86% accuracy in distinguishing patients with amnestic mild cognitive impairment from healthy controls [9], and 70–80% accuracy in distinguishing schizophrenic patients from non-psychiatric controls [10]. In PTSD, Zilcha-Mano et al. [9] were able to discriminate between 51 PTSD individuals and 76 trauma-exposed healthy control subjects with an accuracy of up to 70.6% by using a whole-brain data-driven definition of functional connectivity biomarkers and regularized partial correlations which revealed differences in functional connectivity within executive control network and salience network between the two groups.

DL methods can automatically identify the optimal representation from the raw data without the need for specialized feature engineering. This is achieved by using a hierarchical structure with varying levels of complexity, including the application of consecutive nonlinear transformations to the raw data. An essential aspect of DL that differentiates it from other machine learning methods is that the features are not manually engineered; instead, they are learned from the data, resulting in a more objective and less bias-prone process. Compared with other machine learning methods such as SVM, DL can achieve higher orders of abstraction, complexity and higher classifier accuracy [29, 30], which makes DL more suitable for detecting complex, scattered and subtle patterns in the data [59].

Consistent with our second hypothesis, a few regions make the largest contribution to classification performance: frontoparietal regions (central executive, CEN) and subcortical areas like (median and anterior) cingulate cortex and amygdala. The CEN is associated with the progress of goal-directed behaviors, such as working memory and attention control [60], and it has also been reported as impaired in PTSD [21, 38]. Specifically, CEN functional disruptions are associated with PTSD symptoms of decreased cognitive functioning across multiple domains, as well as emotion under-modulation associated with impaired regulation of limbic structures [61–63]. For instance, a recent study found that in the resting-state, subtype non-differentiated PTSD patients demonstrate reduced CEN convergence, which was associated with decrease orbitofrontal-amygdala connectivity in PTSD, an indicator of reduced prefrontal regulation acting on the resting limbic system [63]. The amygdala is a core area in current neurocircuit models of stress and PTSD [64, 65]. Among its multiple functions, the best known is to encode and extinguish the memory of fearful stimuli [65, 66] so as to direct physiological and behavioral responses to such stimuli. In addition, the amygdala plays an essential role in fear generalization [67], arousal [68] and processing of rewards [69], all of which may be disrupted in PTSD. Exaggerated amygdala activity in response to trauma-related and more generic stimuli is a frequent finding in fMRI studies of PTSD [70, 71]. Recent research has enlarged the functions traditionally ascribed to the cingulate to include emotion [72], pain management [73] and cognitive control [74, 75]. A recent meta-analysis concludes that cingulate plays an important role in emotion and cognitive processing in patients with PTSD [76].

Neuroimaging is still far from becoming a routine tool in clinical psychiatry, mainly because there is still insufficient evidence of diagnostic and prognostic effectiveness. We followed recent recommendations on avoiding methodological issues that may in the past led to overoptimistic results [77–79]. A major challenge in applying machine learning to high-dimensional neuroimaging data is the risk of overfitting, i.e., the learning of irrelevant fluctuations within a dataset that limits generalizability to other datasets. To avoid that, we applied DL technology to conduct a dimension reduction and mitigate the effect of spurious signals. We also tried to minimize the risk of overfitting through the use of region-level features rather than voxel-level data (which are associated with more noise and a higher risk of overfitting) [30]. One limitation is that we only explored topological properties based on the AAL brain atlas. Although AAL is widely accepted in neuroimaging studies, it has drawbacks. Future studies should verify our results using the new brain atlases that are now being used in neuroimaging and machine learning studies, such as the Power 264-region atlas [80] and the Dosenbach’s 160 functional atlas [81]. Another limitation is that our PTSD participants were exposed to a specific traumatic event (an earthquake), which might limit the generalizability of our results. This can be tested by replication using subjects exposed to other traumatic events.

## Conclusion

Despite these limitations, the present study demonstrates DL as an objective and useful classifier which could differentiate pediatric PTSD and HC based on graphic topological measures using resting-state MRI data with promising accuracy. Further, the CEN, parietal gyrus, cingulate cortex, and amygdala provide the greatest contribution to classification performance in DL model, suggesting that investigating these core nodes may give insight into the heterogeneous clinical profiles of individuals with PTSD. Further studies will be needed to assess the clinical applicability of our method.

## Supplementary Information

**Additional file 1.**

## Data Availability

The datasets used and/or analyzed during the current study are available from the corresponding author on reasonable request.

## Abbreviations

PTSD: Posttraumatic stress disorder
HC: Healthy control
DL: Deep learning
SVM: Support vector machines
CEN: Central executive network
PCL: PTSD Checklist
CAPS: Clinician-Administered PTSD Scale
DSM-IV: Diagnostic and Statistical Manual of Mental Disorders, Fourth Edition
FD: Frame-wise displacement
AUC: Area under the curve
AAL: Automated anatomical labeling
