# Supplementary material for: Using deep learning to classify pediatric posttraumatic stress disorder at the individual level
Source: BMC Psychiatry. 2021 Oct 28;21:535. doi: 10.1186/s12888-021-03503-9 (PMC8555083; doi:10.1186/s12888-021-03503-9)
Supplement: Supplementary file 1 — Additional file 1. [file 12888_2021_3503_MOESM1_ESM.docx]

**Additional Information**

# Deep learning model

## Binary preprocessing of raw features

The raw high-dimensional features were represented as a matrix $X\in\mathbb{R}^{m\times d}$ where the element $x_{ij}$ denotes the feature $j$ for subject $i$, and the labels were represented as an $m$-dimensional vector $Y\in{\{0,1\}c}^{m}$, with 1 indicating posttraumatic stress disorder (PTSD) patients and 0 indicating healthy controls (HC). Given the common application of binary input in unsupervised two-layer greedy networks [[1](#_ENREF_1), [2](#_ENREF_2)], we applied a binary transformation to high-dimensional real-valued features resulting in a binary matrix $X\in\left\{ 0,1 \right\}^{m\times d}$. The binary threshold of feature $j$ is calculated as $(m_{0}^{j}+m_{1}^{j})/2$ where $m_{0}^{j}$ denotes the median value of feature $j$ in the HC group and $m_{1}^{j}$ denotes the median value of feature $j$ in the PTSD group. The values of feature$j$ less than or equal to the threshold are assigned 0, and those larger than the threshold are assigned 1. To ensure independence between the training and validation sets, the binary transformation threshold was estimated from the training set and applied to the validation set in each cross-validation loop.

## Network architecture

The core of our classification model is the implementation of the neural network for non-linear dimensionality reduction. A multi-layer neural network generally includes one input layer, several hidden layers, and one output layer. Each layer contains a set of artificial neurons which exactly correspond to the feature representations at different level. The feature representations at different levels are associated by connections between neurons of two consecutive layers: these indicate the strength and direction from input to output, enabling the level-by-level information flow via weighted combination. A non-linear activation function [e.g. ReLU (hidden layer) or Sigmoid (output layer)] is additionally applied between two consecutive layers to handle the potential linear inseparability. In supervised training, the neural network iteratively updates weights by back-propagation to minimize the loss between the network outputs and true labels. Thus deep neural networks can automatically abstract optimal low-dimensional information from raw features via a hierarchical architecture with consecutively decreasing numbers of neurons in each layer. Here we implemented a five-layer neural network for non-linear dimensionality reduction in each model. For the resting-state functional graphic measures model, the number of units in hidden layers were set to 140, 28, 6.

## Network training

Neural network training is performed using a two-step protocol. In the first step, pre-training is implemented via another unsupervised deep learning (DL) model called auto-encoders (AE) with a typical structure comprising encoder and decoder. The architecture of the encoder is similar to a common neural network, which likewise generates low-dimensional representations. Then the decoder utilizes the latent information to reconstruct the original input (a detailed description is given in [[3](#_ENREF_3)]). Without any given labels, AE takes advantage of the inherent pattern in data by minimizing the reconstruction error in an unsupervised manner. Moreover, shallow AEs can be stacked into a deeper structure called stacked autoencoders (SAE), enabling the extraction of higher-order latent representations. In this case, individual AE is greedily trained, and the optimal latent representation of the previous AE is delivered to the next AE to generate a more abstract representation. Here we trained a SAE model with 4 three-layer AEs (1 input layer, 1 hidden layer and 1 output layer) whose architectures conform to the supervised deep neural network described below. Individual AE is taken as having converged when one of the following criteria is met: (1) default 100 epochs are reached; (2) the reconstructed loss measured by mean square error (MSE) for the last 10 epochs is continuously less than 0.001. To weaken the impact of random weight initialization, the training process of SAE was repeated 10000 times. The median values of these fine-tuned weights were transferred to the subsequent deep neural network as initial weights for the next, supervised, step.

In the supervised learning step, a five-layer neural network was trained to fine-tune the weights and biases. The SoftMax function was additionally applied to project the scalar output into probability of binary classes for supervised learning. The training loss estimated by cross-entropy function was optimized with an adaptive moment estimation (Adam) optimizer (momentum parameters = [0.9,0.999], initial learning rate = 0.001). Once the training was complete, the representations in the layer before output layer were extracted as the optimal features in low-dimensional space.

1. *Comparison with other machine learning models*

We compared our approach with three other machine learning models, with the results shown in the Supplementary Table below. Deep learning offers superior specificity at the expense of slightly lower sensitivity, resulting in slightly superior overall accuracy.

| Machine Learning Model | Accuracy  (mean±SD) | Sensitivity  (mean±SD) | Specificity  (mean±SD) |
| --- | --- | --- | --- |
| SVM | 62.6±14.2% | 67.3±20.5% | 58.0±25.0% |
| MLP | 66.1±13.8% | 69.3±22.5% | 63.3±19.4% |
| Random forest | 68.1±14.3% | 68.1±14.3% | 63.3±24.3% |
| Deep learning | 71.2±12.9% | 59.7±21.9% | 82.7±13.9% |

**Supplementary table:** Comparison between different machine learning models

Abbreviations: Support Vector Machine, SVM; Multilayer perceptron, MLP.

**
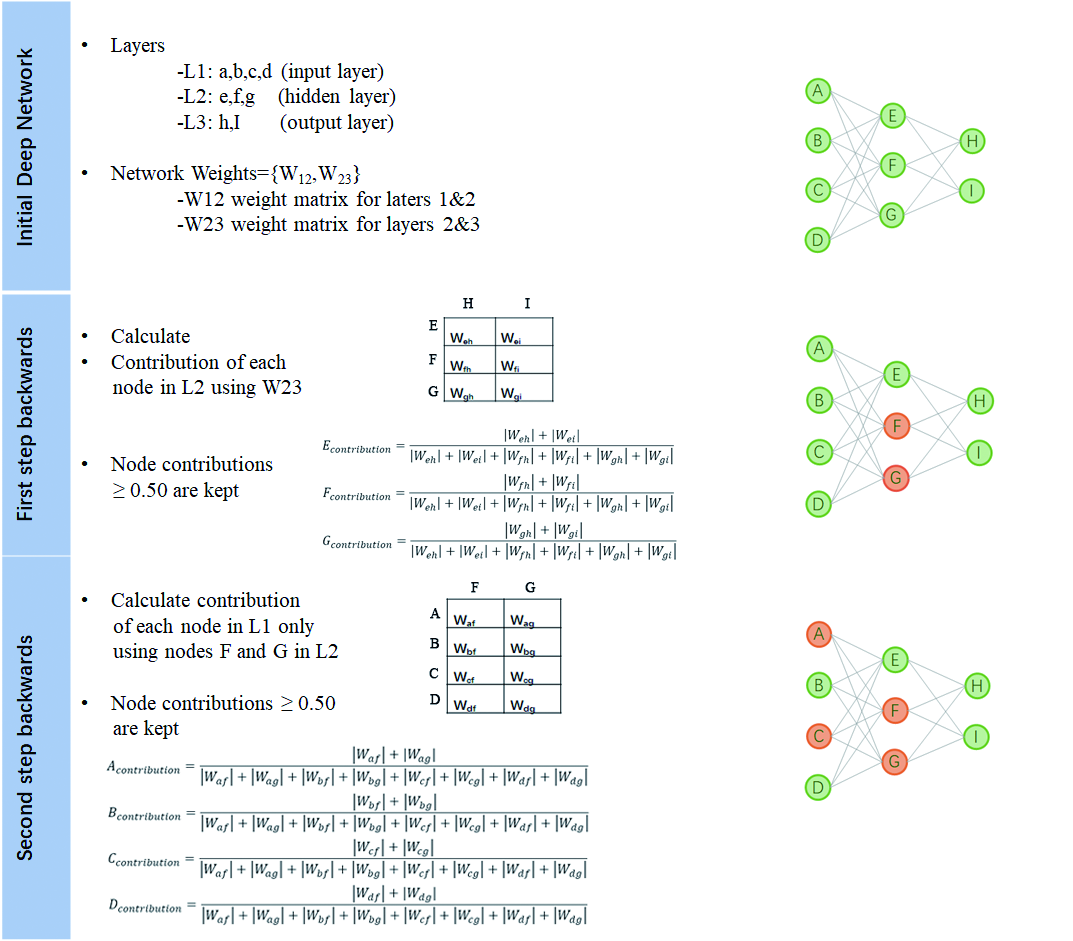
**

**Supplementary Figure:** Example of the procedure used to identify nodes that contribute most to the prediction performance (see [[3](#_ENREF_3)]).

**References**

1. Hinton GE, Salakhutdinov RR. Reducing the dimensionality of data with neural networks. Science (New York, NY). 2006; 313(5786):504-507.

2. Lee H, Grosse R, Ranganath R, Ng A. Convolutional deep belief networks for scalable unsupervised learning of hierarchical representations; 2009.

3. Hazlett HC, Gu H, Munsell BC, Kim SH, Styner M, Wolff JJ, Elison JT, Swanson MR, Zhu H, Botteron KN, et al. Early brain development in infants at high risk for autism spectrum disorder. Nature. 2017; 542(7641):348-351.
